# Supplementary material for: The Gut Microbiome and Alcoholic Liver Disease: Ethanol Consumption Drives Consistent and Reproducible Alteration in Gut Microbiota in Mice
Source: Life (Basel). 2020 Dec 24;11(1):7. doi: 10.3390/life11010007 (PMC7823357; doi:10.3390/life11010007)

Title: The Gut Microbiome and Alcoholic Liver Disease: Ethanol Consumption Drives Consistent and Reproducible Alteration in Gut Microbiota in Mice

Authors: Erick S LeBrun[1], Meghali Nighot[2], Viszwapriya Dharmaprakash[2], Anand Kumar[1], Patrick Chain[1], Chein-Chi Lo[1], and Thomas Y Ma[3]

[1]Biosecurity and Public Health, Los Alamos National Laboratory, Los Alamos, NM 87545

[2]Department of Medicine, Division of Gastroenterology and Hepatology, Penn State College of Medicine, Hershey, PA 17033

[3]Department of Internal Medicine, University of New Mexico School of Medicine, Albuquerque, NM 87131

*Corresponding Author

Keywords: Gut Microbiome, Microbial Ecology, Indicator Species, Bacterial Communities, Mouse Model, Alcohol Effects, Alcoholic Liver Disease, Microbiome, Ethanol Induced Liver Disease, Leaky Gut

Supplementary Figures

Figure S1: Schematic of experimental design.

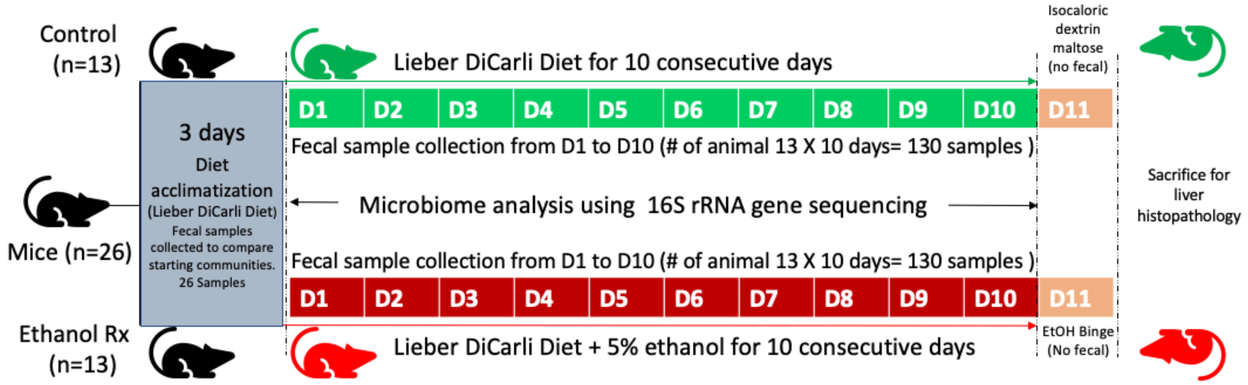

Figure S2: Boxplots of alpha diversity scores. A) Shannon diversity (H) B) Simpson diversity (D).

A)

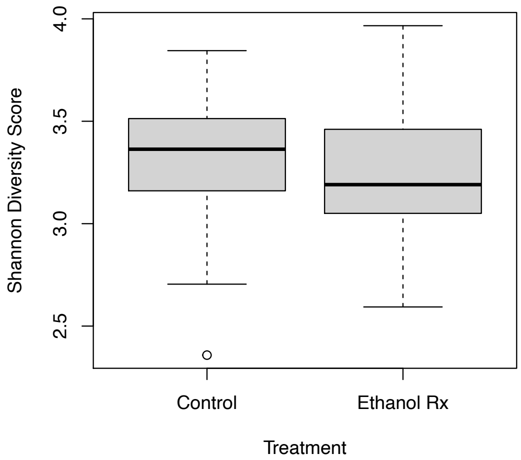

B)

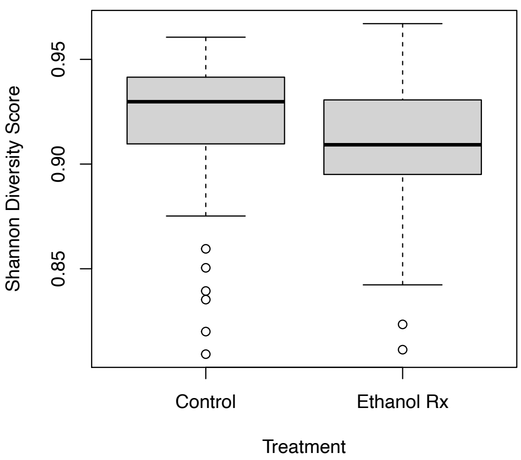

Figure S3: Beta dispersion of Controls vs. Ethanol Treatments. A) PCoA plot of beta dispersion. B) Boxplot of distance to centroid values.

A)

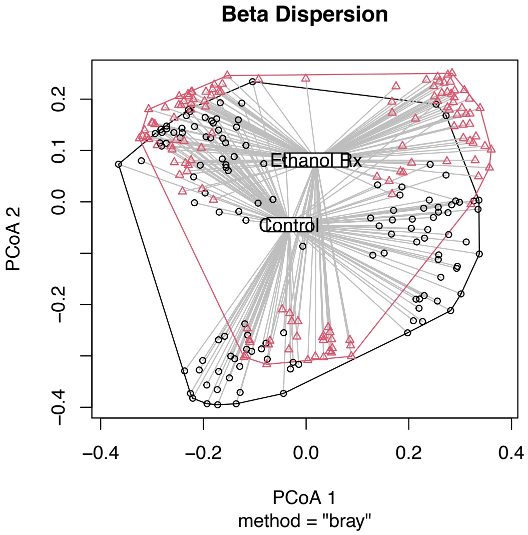

B)

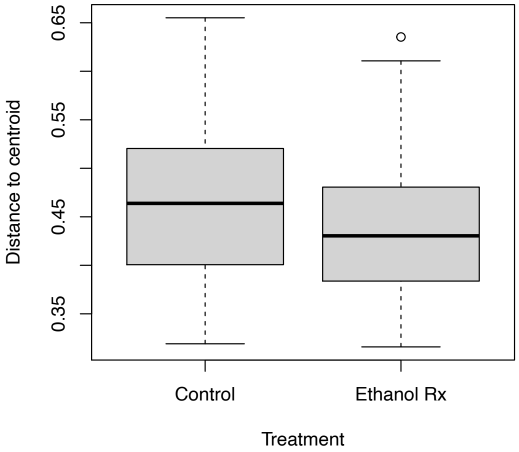

Figure S4: TITAN2 plots. A) Significant taxa identified by TITAN2. Dots represent changepoints with bars representing 95% confidence intervals. Z- scores are decreasing in abundance while Z+ scores are increasing. B) Community-level sums of taxon-specific change.

A)

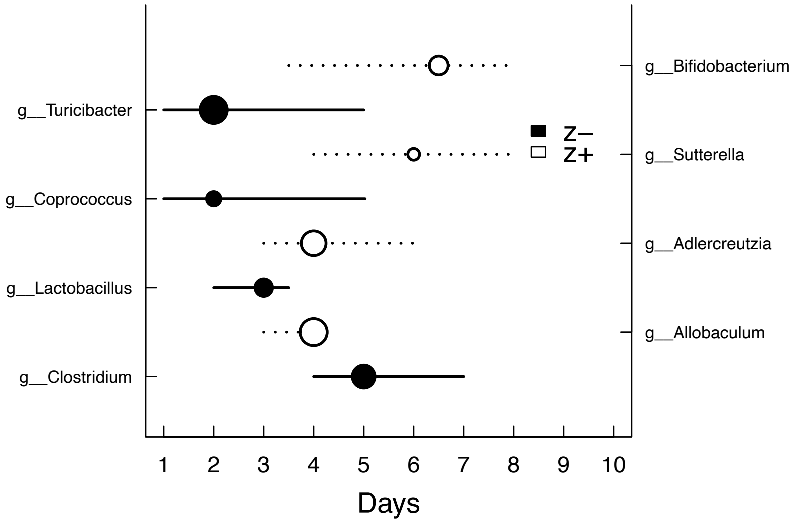

B)

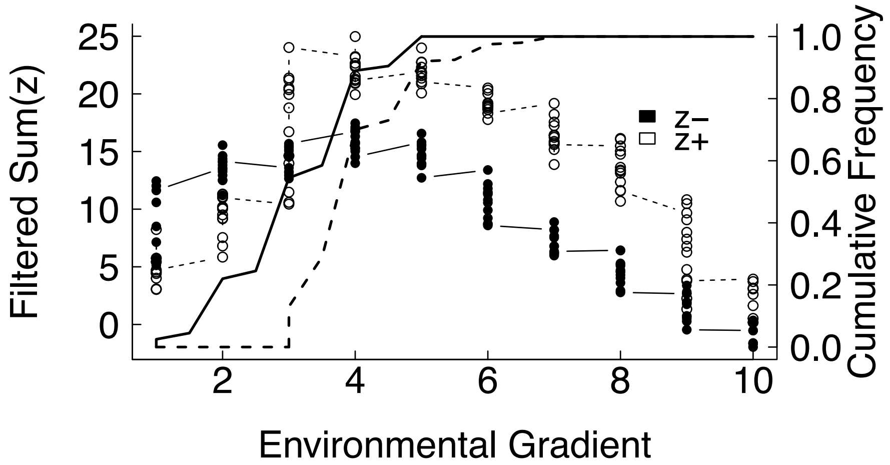

Figure S5: PERMANOVA and ANOSIM statistics and p-values for *k* clusters.

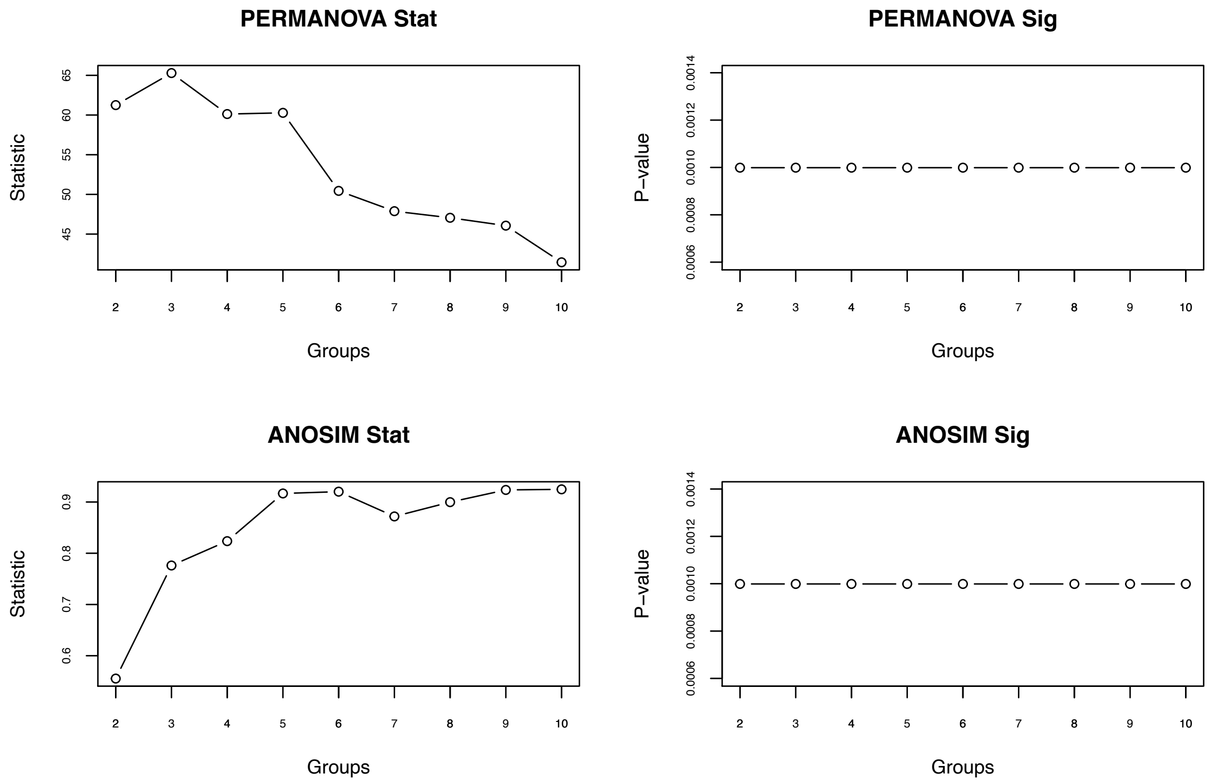

Figure S6: Three alternative views in NMDS of *k*=3 clusters annotated with Experiment and Treatment metadata.

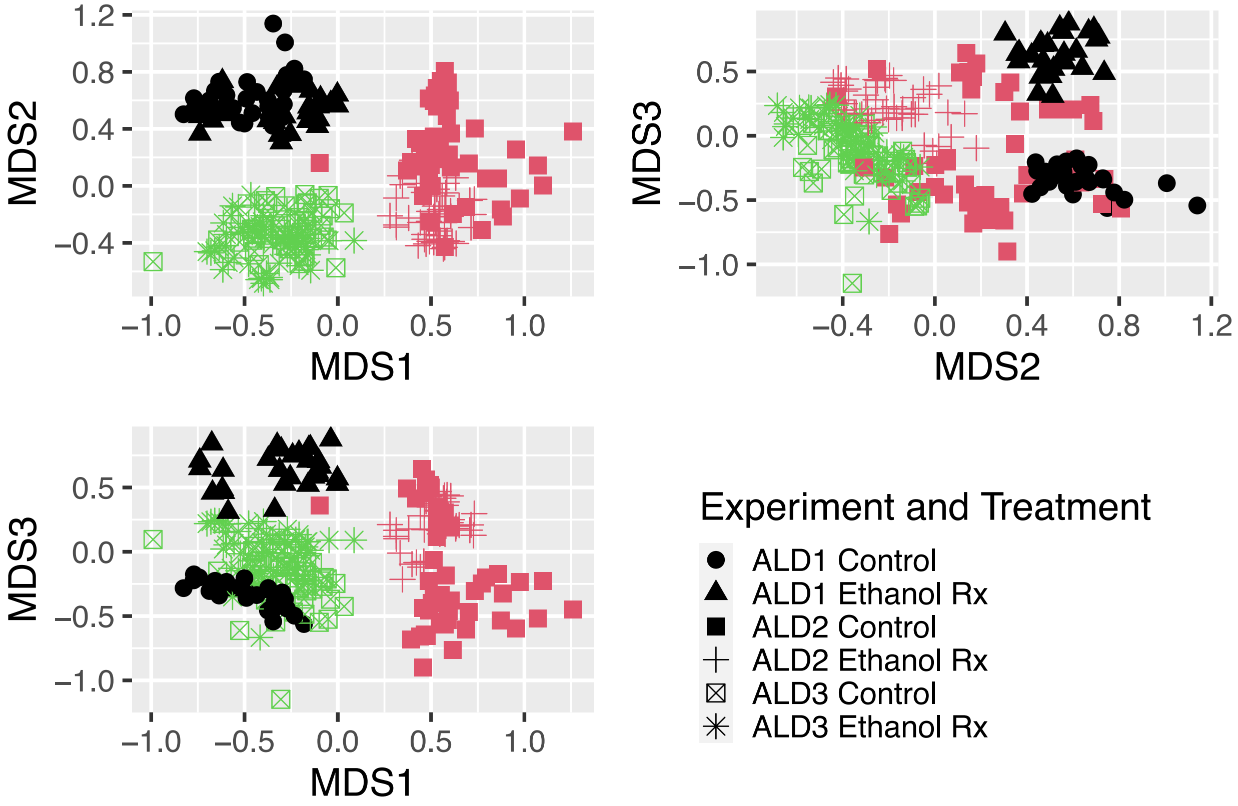

Figure S7: Three alternative views in NMDS of k=4 clusters annotated with Experiment and Treatment metadata.

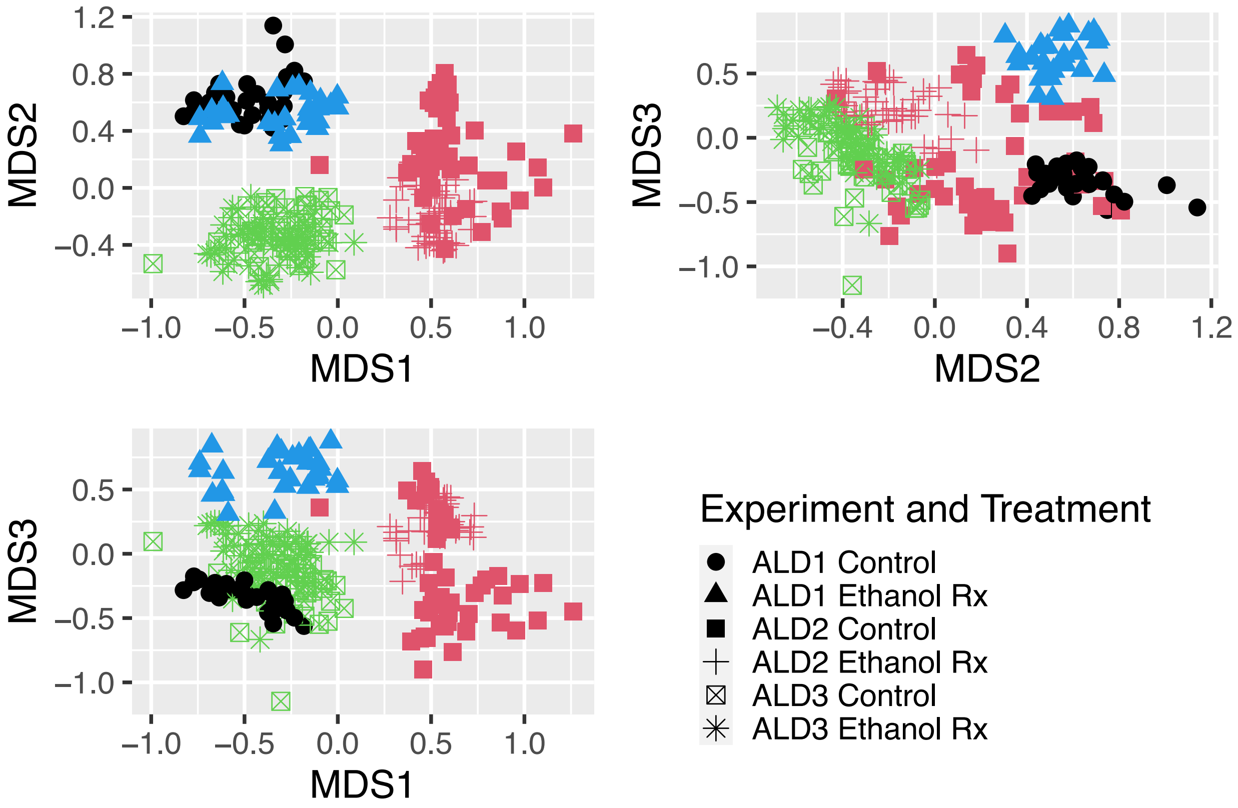

Figure S8: Three alternative views in NMDS of *k*=5 clusters annotated with Experiment and Treatment metadata.

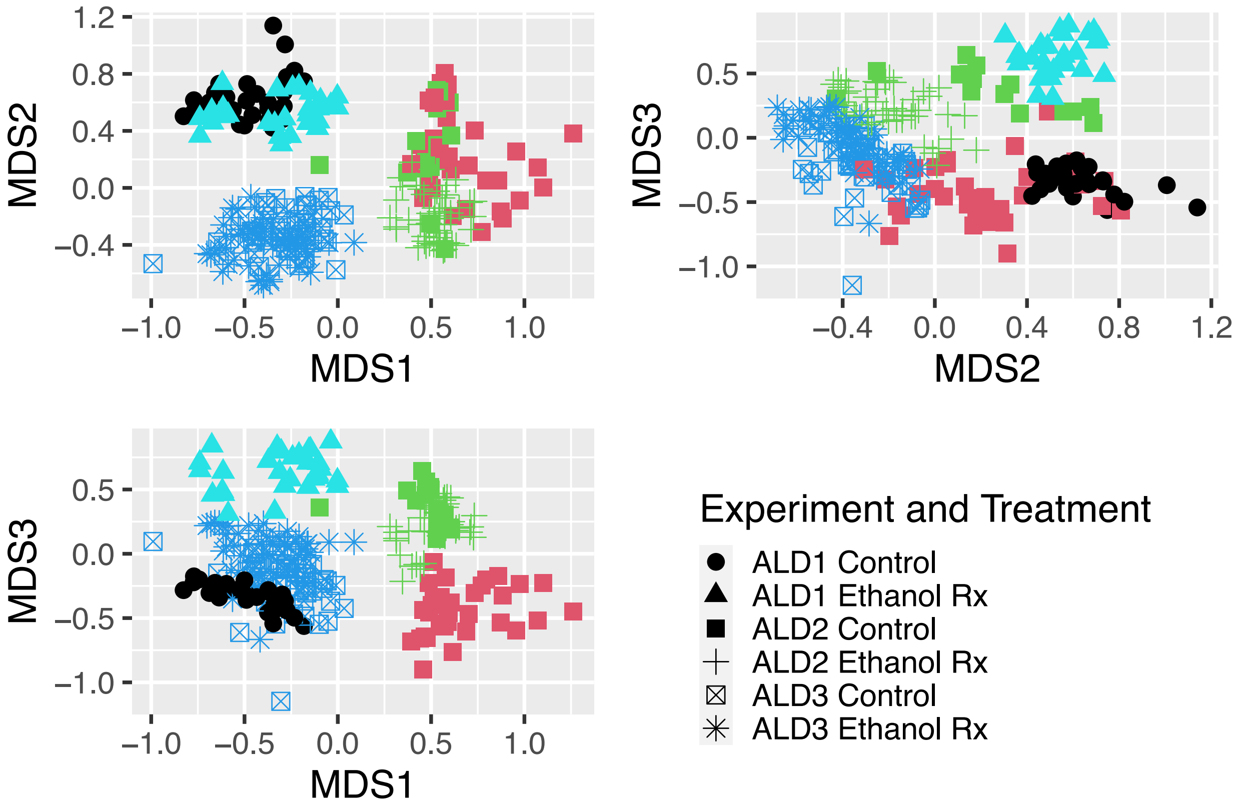

Figures S9 – S34: Mean relative abundance plots of significant genera in Controls and Ethanol treatments from day 4 through day 13. Figure S32, *Akkermansia* was investigated despite not being significant.

Figure S9:

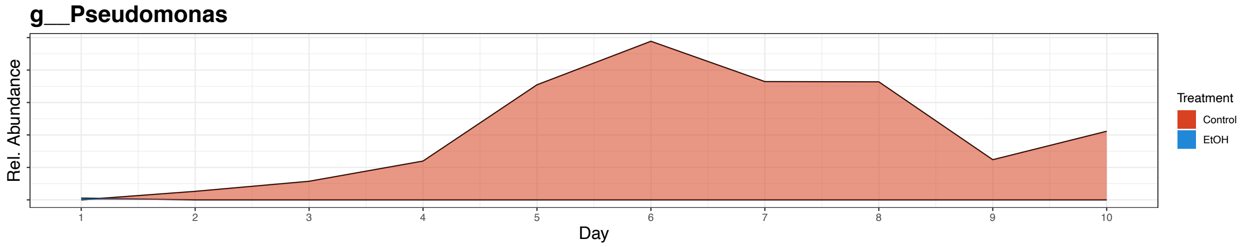

Figure S10:

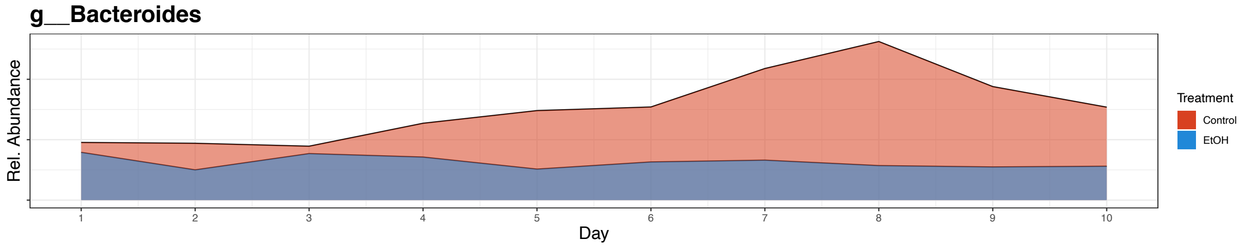

Figure S11:

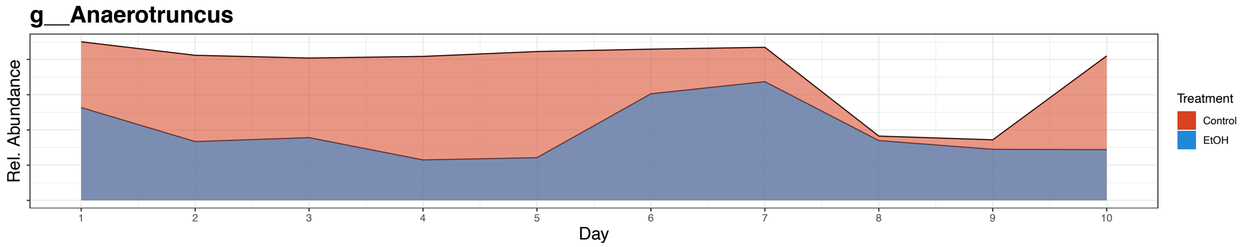

Figure S12:

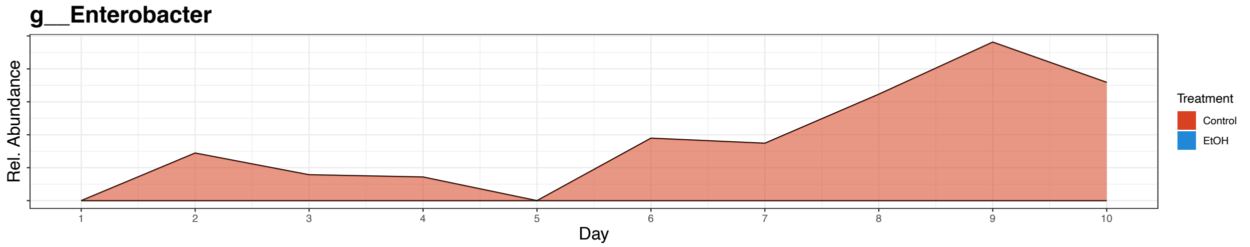

Figure S13:

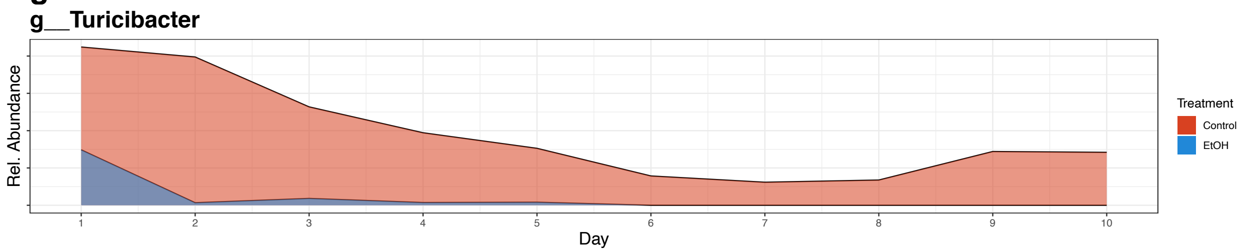

Figure S14:

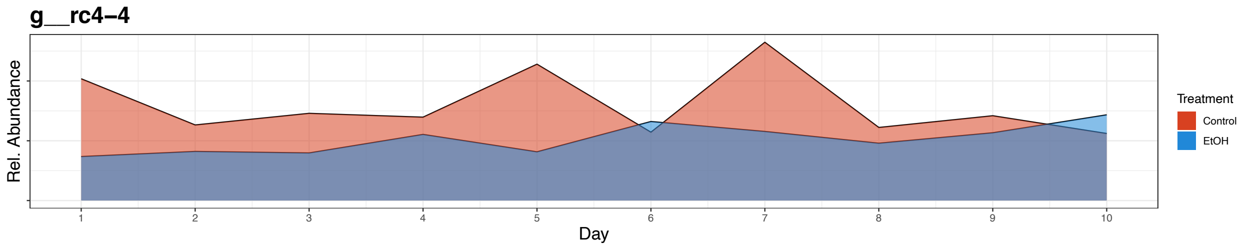

Figure S15:

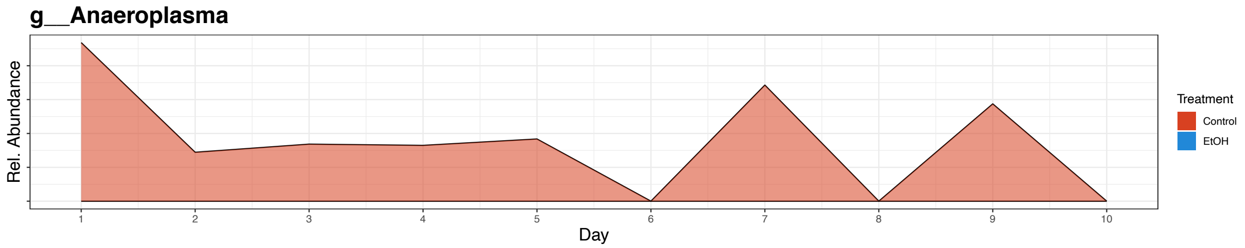

Figure S16:

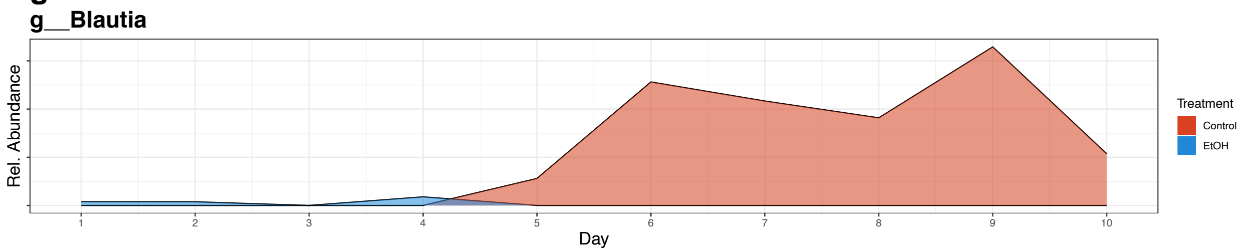

Figure S17:

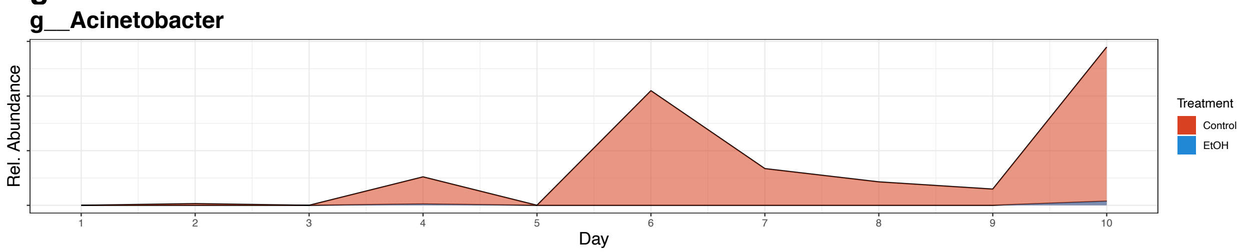

Figure S18:

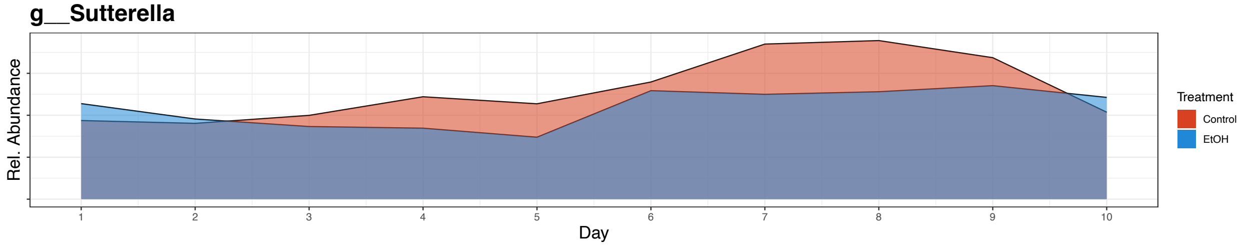

Figure S19:

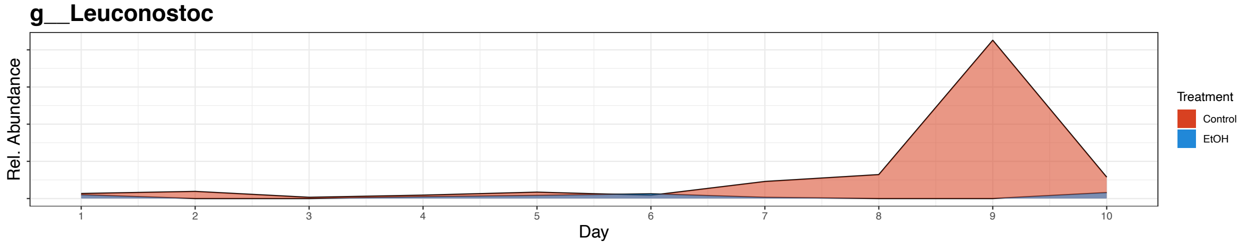

Figure S20:

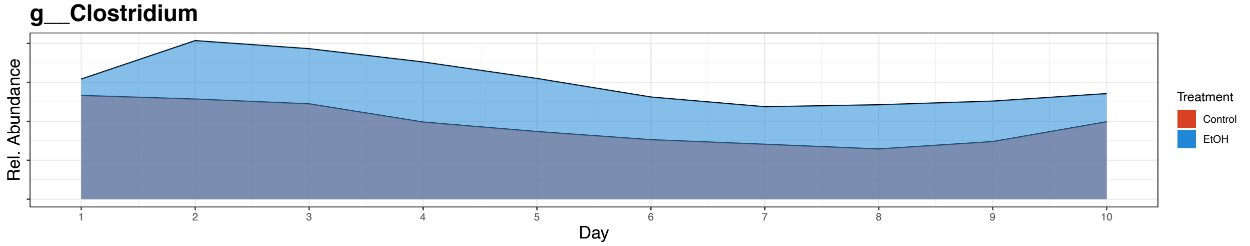

Figure S21:

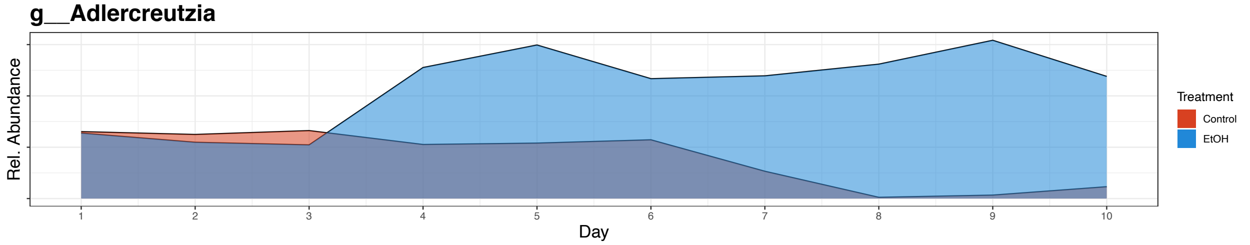

Figure S22:

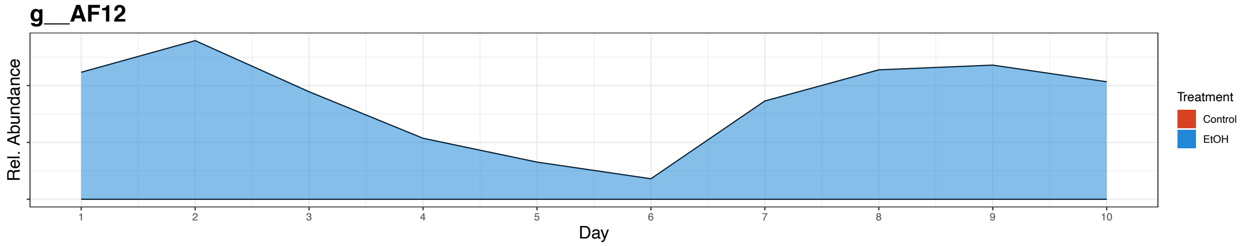

Figure S23:

g__Bilophila

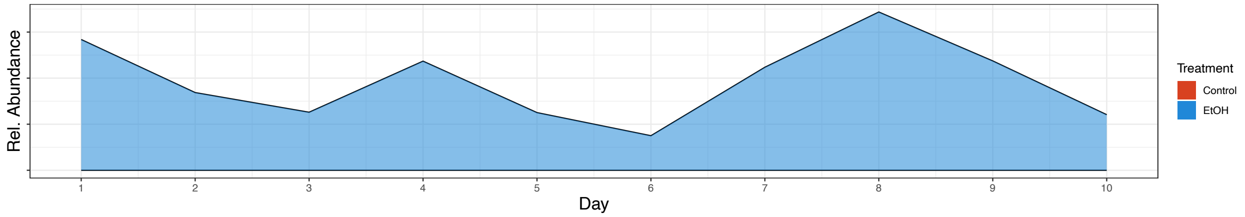

Figure S24:

g__Helicobacter

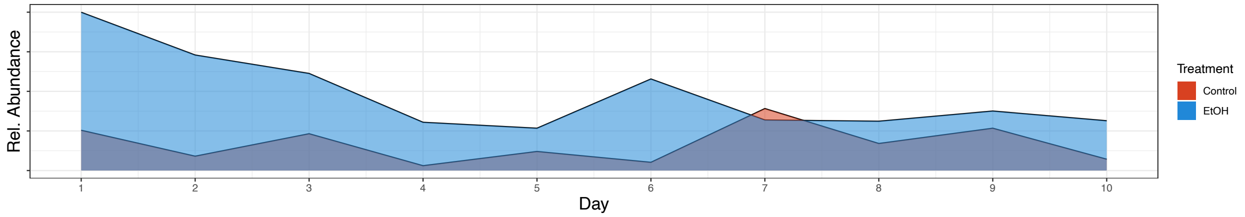

Figure S25:

g__Prevotella

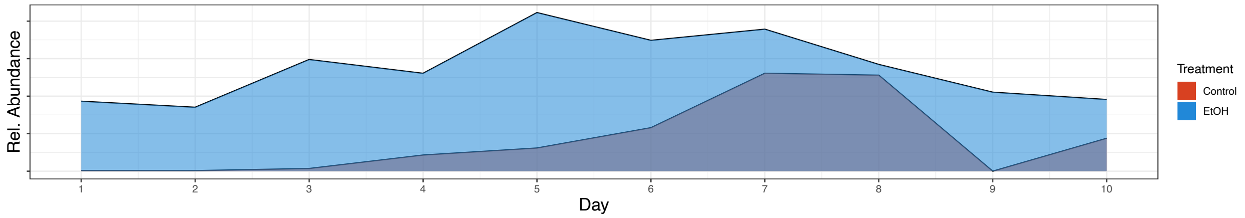

Figure S26:

g__Enterococcus

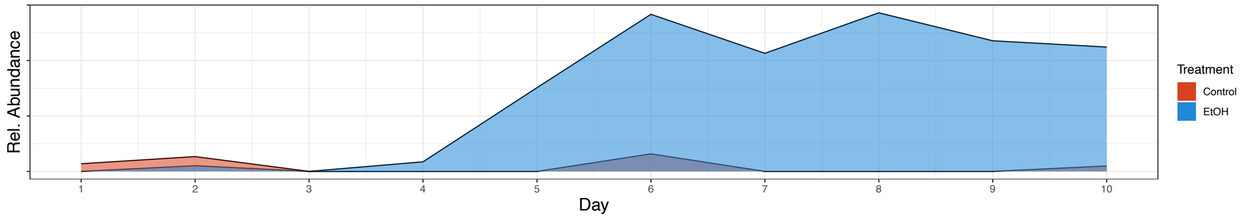

Figure S27:

g__Desulfovibrio

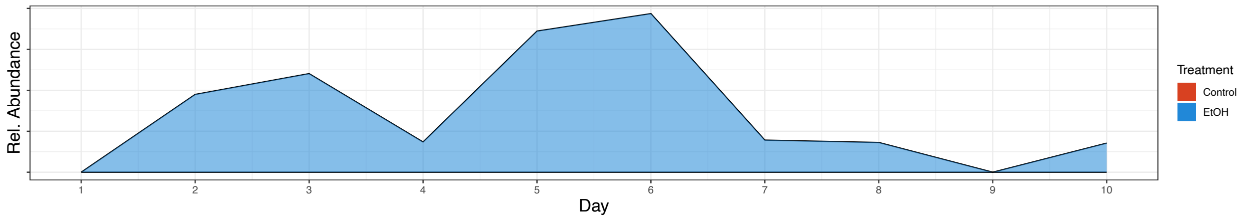

Figure S28:

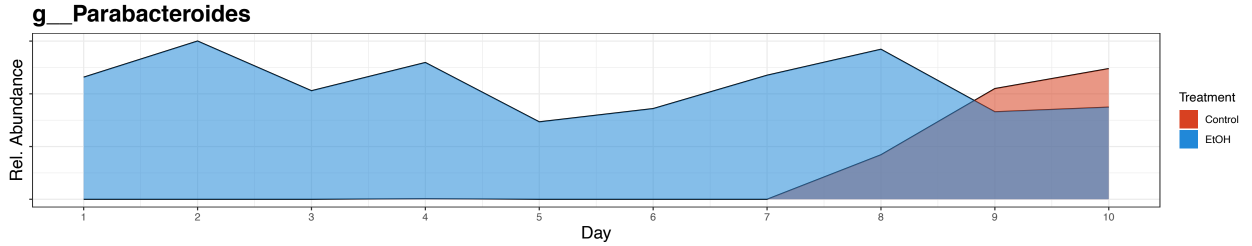

Figure S29:

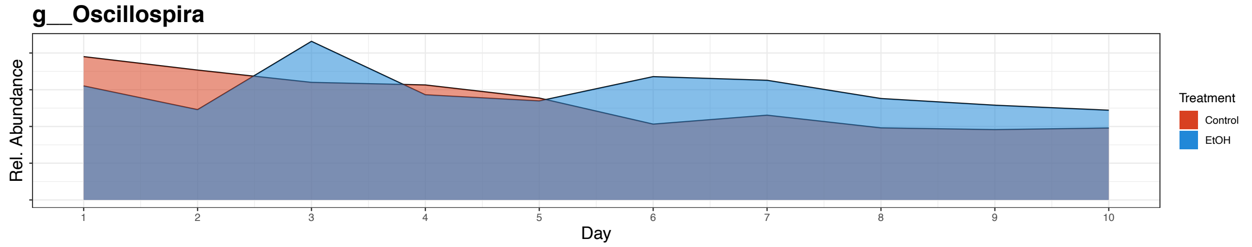

Figure S30:

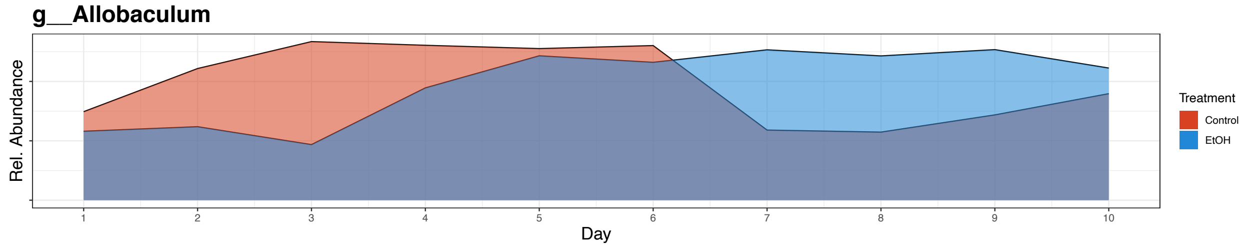

Figure S31:

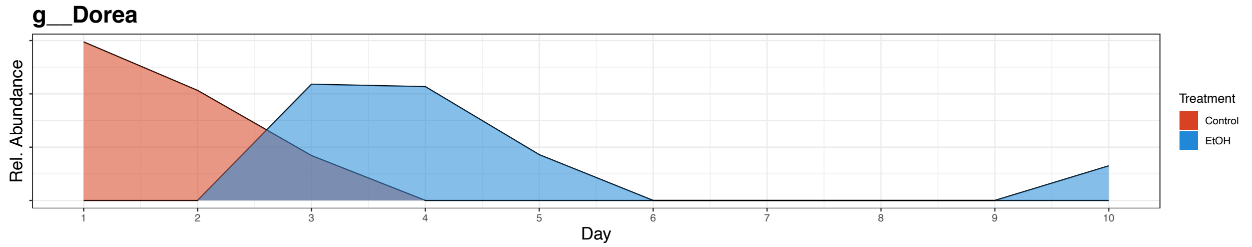

Figure S32:

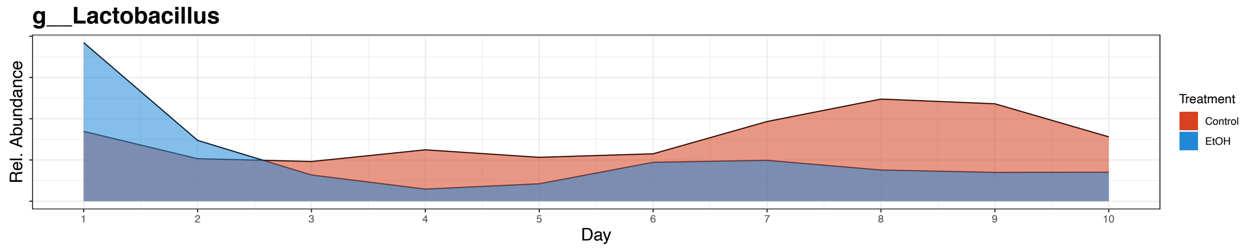

Figure S33:

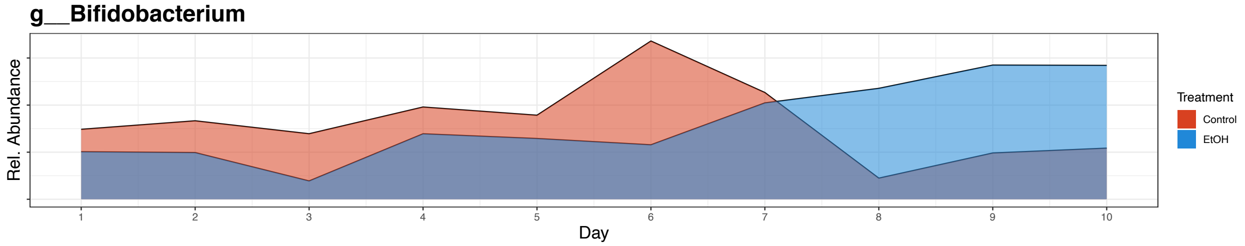

Figure S34:

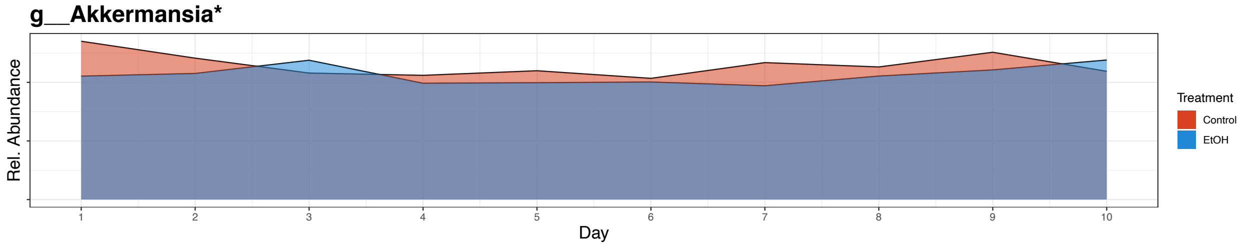

Supplement: Supplementary file 1 [file life-11-00007-s001.zip › LeBrun_et_al_sup_figures_mdpi.pdf]
