# Supplementary material for: The Gut Microbiome and Alcoholic Liver Disease: Ethanol Consumption Drives Consistent and Reproducible Alteration in Gut Microbiota in Mice
Source: Life (Basel). 2020 Dec 24;11(1):7. doi: 10.3390/life11010007 (PMC7823357; doi:10.3390/life11010007)
Supplement: Supplementary file 1 [file life-11-00007-s001.zip › LeBrun_et_al_sup_methods_mdpi.pdf]

Title: The Gut Microbiome and Alcoholic Liver Disease: Ethanol Consumption Drives Consistent and Reproducible Alteration in Gut Microbiota in Mice

Authors: Erick S LeBrun[1], Meghali Nighot[2], Viszwapriya Dharmaprakash[2], Anand Kumar[1], Chien-Chi Lo[1], Patrick SG Chain[1]**, and Thomas Y Ma[2,3,*]

[1]Biosecurity and Public Health, Los Alamos National Laboratory, Los Alamos, NM 87545

[2]Department of Medicine, Division of Gastroenterology and Hepatology, Penn State College of Medicine, Hershey, PA 17033

[3]Department of Internal Medicine, University of New Mexico School of Medicine, Albuquerque, NM 87131

*Corresponding Author: thomasma@pennstatehealth.psu.edu

** Co-corresponding Author: pchain@lanl.gov

Keywords: Gut Microbiome, Microbial Ecology, Indicator Species, Bacterial Communities, Mouse Model, Alcohol Effects, Alcoholic Liver Disease, Microbiome, Ethanol Induced Liver Disease, Leaky Gut

Supplemental Methods

Sequencing and Processing

For first round PCR using KAPA HiFi HotStart Ready Mix (KAPA Biosystems, Inc., South Africa) a single hot start PCR step of 95ºC for 3 minutes was performed followed by 20 cycles of denaturation at 95ºC for 30 seconds, annealing at 55ºC for 30 seconds, and elongation at 72ºC for 30 seconds, finishing with a single step of extension at 72ºC for 5 minutes prior to holding at 4ºC.

For second round PCR to add Nextera XT v2 indexes (Illumina, CA, USA), an initial step at 95ºC for 3 minutes was performed followed by 8 cycles of denaturation at 95ºC for 30 seconds, annealing at 55ºC for 30 seconds, elongation 72ºC for 30 seconds, and finally by a single extension step at 72ºC for 5 minutes prior to holding at 4ºC.

Amplicons were cleaned using AMPure XP beads (Beckman Coulter, Cat. #A63881). A no template control was processed but did not show a band in the V4 amplicon region and was not further processed. Concentration of the amplicon pool was obtained using the Qubit dsDNA HS Assay (ThermoFisher Scientific, Cat. #Q32854). Average size of the library was determined by the Agilent High Sensitivity DNA Kit (Agilent, Cat. #5067-4626). Further accurate library quantification was performed using the Library Quantification Kit – Illumina/Universal Kit (KAPA Biosystems, Cat. #KK4824).

For QIIME2/DADA2 pipeline processing, sequencing reads are set by default to be trimmed at a quality score threshold of q=2. The first 10 bases of reads were trimmed and reads were truncated at position 250 for forward reads and 300 for reverse reads

due to standard decreases in quality. If the resulting reads were shorter than 250 bp, they were discarded. The DADA2 algorithm was then applied to the data set to denoise the reads (DADA2 corrects substitution and indel errors and infers sequence variants). After denoising, forward and reverse reads were joined to obtain full length, denoised sequences by aligning the denoised forward reads with the reverse-complement of the corresponding denoised reverse reads. ASVs were then constructed from unique sequences. ASVs were further curated in the QIIME2-DADA2 pipeline by removing chimeras from each sample. Reads were then classified as outlined in the primary manuscript.
